# Reading and writing habits compensate for aging effects in speech connectedness

**DOI:** 10.1038/s41539-022-00129-8

**Published:** 2022-06-08

**Authors:** Bárbara L. C. Malcorra, Natália B. Mota, Janaina Weissheimer, Lucas P. Schilling, Maximiliano A. Wilson, Lilian C. Hübner

**Affiliations:** 1 School of Humanities, Graduate Course in Linguistics, Pontifical Catholic University of Rio Grande do Sul (PUCRS), Porto Alegre, RS Brazil; 2 Institute of Psychiatry, Federal University of Rio de Janeiro (UFRJ), Rio de Janeiro, RJ Brazil; 3 Department of Physics, Federal University of Pernambuco (UFPE), Recife, PE Brazil; 4 Brain Institute, Department of Foreign Languages and Literatures, Federal University of Rio Grande do Norte (UFRN), Natal, RN Brazil; 5 National Council for Scientific and Technological Development (CNPq), Brasília, DF Brazil; 6 School of Medicine, Graduate Course in Medicine and Healthy Sciences, Pontifical Catholic University of Rio Grande do Sul (PUCRS), Porto Alegre, RS Brazil; 7 School of Medicine, Graduate Course in Biomedical Gerontology, Pontifical Catholic University of Rio Grande do Sul (PUCRS), Porto Alegre, RS Brazil; 8 Brain Institute of Rio Grande do Sul (InsCer), Pontifical Catholic University of Rio Grande do Sul (PUCRS), Porto Alegre, RS Brazil; 9 Centre interdisciplinaire de recherche en réadaptation et intégration sociale (CIRRIS), Département de réadaptation, Université Laval, Québec City, QC Canada

**Keywords:** Education, Education, Cognitive ageing

## Abstract

We investigate the association of short- and long-range recurrences (speech connectedness) with age, education, and reading and writing habits (RWH) in typical aging using an oral narrative production task. Oral narrative transcriptions were represented as word-graphs to measure short- and long-range recurrences. Speech connectedness was explained by the combination of age, education, and RWH, and the strength of RWH’s coefficient reflects the aging effect.

Formal education and reading and writing habits (RWH) seem to protect from the cognitive decline associated with typical aging. Aging studies have reported positive effects of education in the production of main ideas and cohesive links^1–3^ and of RWH on executive functions, attention, memory^4,5^ and language^6^. A recent longitudinal study verified that RWH prevents long-term decline in cognitive functioning^7^.

Recently, a graph-theoretical-based approach applied to spontaneous narratives succeeded in representing speech patterns associated with cognitive changes in different contexts (from typical development to atypical decline)^8–24^. It is possible to measure short or long-range recurrences (connectedness) in a narrative represented as a word-graph. Speech connectedness, which is a quantitative measure of the relationship between the elements in a text that determine its unity, increases in parallel with cognitive development, with an important role played by formal education^8,10^. As soon as a child starts to read, the oral narrative structure changes from a short to a long-range recurrence pattern, increasing connectedness^8^. Low speech connectedness is linked to cognitive decline associated with schizophrenia^9,11,15^, attention deficit hyperactivity disorder^24^, and Alzheimer’s disease (AD)^17^, but little is known about the impact of formal education during a typical cognitive decline in healthy aging. In healthy children and adults (up to 60 years old), education explained better than age the relationship between speech connectedness and development^8^. While short-range recurrences diminish as the child starts reading, speech connectedness matures more slowly, at the end of high school^8^. For more information, please read Supplementary Note 1.

Considering the growing life expectancy worldwide and the increase in dementia rates associated with low education and socioeconomic status (SES)^25^, word-graph analysis can shed light on the effects of these socioeconomic aspects on cognition. Formal education and the frequency of RWH can be grouped under the concept of cognitive reserve which establishes the theory that activities that stimulate the brain are linked to an increase in brain resilience to cope with changes in cognitive processing resulting from typical and atypical aging^26–29^. The present study aims to investigate the effect of education and RWH on the oral production of narratives by typical adults and older adults. We hypothesized that there would be a protective effect of education and RWH in the production of oral narratives of typical adults and older adults, which would be verified by an attenuation in the increase in short-range recurrences and in the decrease in long-range recurrences.

Narratives from 118 healthy individuals (mean age 68.71 ± 6.44 years) with low education (mean years of 9.99 ± 5.73) were represented as word-graphs, and short-range recurrence (repeated edges [RE]) and long-range recurrence (connectedness – largest connected component [LCC] and largest strongly connected component [LSC]) were calculated. Graph analysis revealed that age correlated positively with RE and negatively with connectedness (LCC). The correlations lost significance when corrected for education and RWH. Education correlated positively with LCC. The correlation remained significant when corrected for age, but lost significance when corrected for RWH. RWH correlated negatively with RE and positively with LCC, and both remained significant when corrected for age, but lost significance when corrected for education (Supplementary Table 2). Importantly, age correlated negatively with education (*R* = –0.22, *p* = 0.016), but not significantly with RWH (*R* = –0.15, *p* = 0.095), and education correlated positively with RWH (*R* = 0.58, *p* ≤ 0.001), as expected.

There were significant canonical correlations between the combination of age, education, and RWH with the three graph attributes. This explained 16% of the variance (Fig. 1). LCC and RWH had higher coefficients and co-varied in the same direction (the higher the RWH, the higher the speech connectedness). Long-range recurrence (LCC and LSC) co-varied in the same direction as RWH and education, and in opposition to age, while RE co-varied with age and in opposition with RWH and education (Fig. 1).

**Fig. 1:**
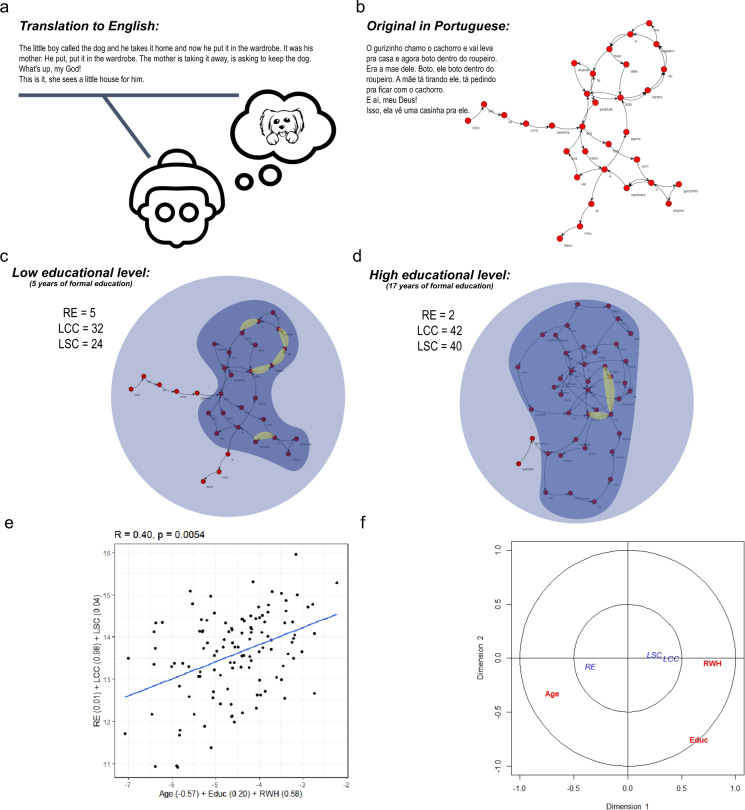
Speech graph analysis of oral narratives during typical aging. **a** Illustrative example of the speech collection protocol. **b** From text to graphs. **c** Low educational status example. **d** High educational status example. **e** Canonical correlation of sets 1 and 2. *R* and *p* values in the title, and the canonical coefficient on the *x* and *y* axes. **f** Representation of each variable coefficient on both canonical dimensions (in blue the speech graph attributes, in red social factors and age, in yellow the repeated edges).

As predicted, advancing age led to increased RE and reduced connectedness, making oral discourse more repetitive and less connected. This relationship lost statistical significance when corrected for education and RWH, suggesting a protective effect of reading on cognition in a low educational level population. This is in accordance with the described dynamic of short- and long-range recurrence during typical development and the association with formal education^8^, which reveals an interesting pattern of speech connectedness across the lifespan. It shows that short-range recurrences that decrease during children’s emerging literacy increase with advancing age. Conversely, the ability to produce long-range recurrences and a well-connected narrative, which increases over school years, decreases in older adults.

In addition, the combination of age, education, and RWH was associated with graph attributes (RE, LCC, and LSC). Therefore, short- and long-range recurrences were only explained by the combination of age, education, and RWH and not by any of these variables in isolation. Interestingly, the strength of RWH’s coefficient compensated for the aging effect, stressing the protective effect of reading and writing throughout life.

These results are in accordance with the concept of cognitive reserve^26^, which establishes that activities that stimulate cognition are linked to an increase in resilience to changes in cognitive processing in typical aging. Furthermore, the more the older adults read books, the higher their levels of verbal fluency^5^, and the higher their phonemic verbal fluency scores^6^. A longitudinal study verified that older adults with higher RWH were less likely to develop cognitive decline^7^. Thus, the present study seems to evidence the positive effects of RWH on connected speech performance, as measured in oral narrative production, in typical older adults, although this could not be taken as a causality effect. In such a scenario, the stimulation of RWH could mitigate the impact of low education and increasing aging rates mainly in low-income and low-literacy adulthood and aging.

Alternatively, as older participants were also less educated, their low connected narratives could reflect a reduced vocabulary and language knowledge. Therefore, future studies should analyze the impact of general vocabulary together with spontaneous narratives produced longitudinally on mediating these effects in typical aging. Also, we should remain cautious with computational approaches to analyze human behavior, as we still need to better understand these markers in representative and larger samples^14,23^. Although considering the limitations, such as the higher proportion of female participants, our findings bring important implications for the maintenance of cognitive activity in maturity. Firstly, cognitive activity can preserve the individual’s quality of life. Secondly, it may cope with the prevalence of neurodegenerative diseases, such as AD, especially in low-income countries, where functional illiteracy is still a bottleneck^30^. Finally, preventive measures and policies to maintain cognitive efficiency seem to be more cost-effective as compared to the remediation of the social impacts on health systems caused by the increase of dementia worldwide.

## Methods

### Participants

We collected narratives from 118 healthy individuals (51–82 years old) (Supplementary Table 1), predominantly with low educational level and low to middle-low SES. The study was approved by the institutional Research Ethics Committee (560.073, CAAE registry number 21006913.0.0000.5336) and participants gave written informed consent.

### Narrative task

Participants performed an oral narrative task based on seven pictures (“The dog story”)^31^. The pictures remained available for the participants while telling the story. There was no time limit for the task. Speech samples were audio-recorded and transcribed for analysis.

### Graph analysis procedure

We represented the oral narrative transcriptions as a word-trajectory graph using the Speech Graphs software^9^. To control verbosity, we analyzed the narratives using a moving window of a fixed word length (30 words) with a step of one word. Three connectedness attributes were calculated: (1) RE, defined as the sum of all edges linking the same pair of nodes; (2) the number of nodes in the LCC, defined as the largest set of nodes directly or indirectly linked by some path; and (3) the number of nodes in the LSC, defined as the largest set of nodes directly or indirectly linked by reciprocal paths, so that all the nodes in the component are mutually reachable. We considered RE as short-range and LCC and LSC as long-range speech connectedness.

### Statistical analyses

The data were not normally distributed (Shapiro–Wilk test). We used a nonparametric test, Spearman correlations, to assess the association between age, education, and frequency of RWH with RE, LCC, and LSC. We corrected the significance level by using the Bonferroni test for three comparisons (*α* = 0.0166). Then, we performed partial correlations and calculated canonical correlations to associate two sets of variables. The first set contained age, education, and RWH, while the second set contained the graph attributes: RE, LCC, and LSC. Variance inflation factor values were within acceptable ranges (<10), suggesting the absence of multicollinearity. We used sets of measures with conditioning numbers lower than 30. All the analyses were performed in RStudio 4.1.0^32^.

### Reporting summary

Further information on research design is available in the Nature Research Reporting Summary linked to this article.

## Supplementary information

Reporting Summary

Supplementary information

## Data Availability

All the data are available in Supplementary Table 3.

## References

[1] le Dorze G, Bédard C (1998). Effects of age and education on the lexico-semantic content of connected speech in adults. J. Commun. Disord..

[2] Mackenzie C (2000). The relevance of education and age in the assessment of discourse comprehension. Clin. Linguist. Phonetics.

[3] Mackenzie C, Brady M, Norrie J, Poedjianto N (2007). Picture description in neurologically normal adults: concepts and topic coherence. Aphasiology.

[4] Pawlowski J (2012). The influence of reading and writing habits associated with education on the neuropsychological performance of Brazilian adults. Read. Writ..

[5] Sörman DE, Ljungberg JK, Rönnlund M (2018). Reading habits among older adults in relation to level and 15-year changes in verbal fluency and episodic recall. Front. Psychol..

[6] Tessaro B (2020). Verbal fluency in Alzheimer’s disease and mild cognitive impairment in individuals with low educational level and its relationship with reading and writing habits. Dement. Neuropsychologia.

[7] Chang, Y.-H., Wu, I.-C. & Hsiung, C. A. Reading activity prevents long-term decline in cognitive function in older people: evidence from a 14-year longitudinal study. *Int. Psychoger.* **33**, 63–74 (2021). 10.1017/S1041610220000812 PMC8482376 32498728

[8] Mota NB, Sigman M, Cecchi G, Copelli M, Ribeiro S (2018). The maturation of speech structure in psychosis is resistant to formal education. npj Schizophrenia.

[9] Mota, N. B., Furtado, R., Maia, P. P. C., Copelli, M. & Ribeiro, S. Graph analysis of dream reports is especially informative about psychosis. *Sci. Rep.* **4**, 3691 (2014). 10.1038/srep03691 PMC3892182 24424108

[10] Mota NB (2016). A naturalistic assessment of the organization of children’s memories predicts cognitive functioning and reading ability. Mind Brain Educ..

[11] Palaniyappan L (2019). Speech structure links the neural and socio-behavioural correlates of psychotic disorders. Prog. Neuro-Psychopharmacol. Biol. Psychiatry.

[12] Mota NB (2020). Verbal short‐term memory underlies typical development of “thought organization” measured as speech connectedness. Mind Brain Educ..

[13] Mota, N. B. et al. Speech graphs provide a quantitative measure of thought disorder in psychosis. *PLoS ONE* **7**, e34928 (2012). 10.1371/journal.pone.0034928 PMC3322168 22506057

[14] Mota NB (2021). Commentary on “Investigating the diagnostic utility of speech patterns in schizophrenia and their symptom associations”: the current need for the harmonization of speech elicitation protocols in basic and applied science. Schizophrenia Res..

[15] Mota, N. B., Copelli, M. & Ribeiro, S. Thought disorder measured as random speech structure classifies negative symptoms and schizophrenia diagnosis 6 months in advance. *npj Schizophrenia* **3**, 18 (2017). 10.1038/s41537-017-0019-3 PMC5441540 28560264

[16] Lemke CE (2021). The effects of early biliteracy on thought organisation and syntactic complexity in written production by 11-year-old children. Lang. Teach. Res. Q..

[17] Malcorra BLC (2021). Low speech connectedness in Alzheimer’s disease is associated with poorer semantic memory performance. J. Alzheimer’s Dis..

[18] Pinheiro S (2020). The history of writing reflects the effects of education on discourse structure: implications for literacy, orality, psychosis and the axial age. Trends Neurosci. Educ..

[19] Bertola L (2014). Graph analysis of verbal fluency test discriminate between patients with Alzheimer’s disease, mild cognitive impairment and normal elderly controls. Front. Aging Neurosci..

[20] Spencer TJ (2021). Lower speech connectedness linked to incidence of psychosis in people at clinical high risk. Schizophrenia Res..

[21] Morgan, S. E. et al. Natural Language Processing markers in first episode psychosis and people at clinical high-risk. *Transl. Psychiatry* **11**, 630 (2021). 10.1038/s41398-021-01722-y PMC8669009 34903724

[22] Corcoran CM (2021). Commentary on “Lower speech connectedness linked to incidence of psychosis in people at clinical high risk”: the promise of graph theory and network neuroscience. Schizophrenia Res..

[23] Palaniyappan L (2021). More than a biomarker: could language be a biosocial marker of psychosis?. npj Schizophrenia.

[24] Coelho, R. M. et al. Network analysis of narrative discourse and attention-deficit hyperactivity symptoms in adults. *PLoS ONE* **16**, e0245113 (2021). 10.1371/journal.pone.0245113 PMC8026017 33826632

[25] Livingston, G. et al. Dementia prevention, intervention, and care: 2020 report of the Lancet Commission. *Lancet* **396**, 413–446 (2020). 10.1016/S0140-6736(20)30367-6 PMC7392084 32738937

[26] Stern Y (2020). Whitepaper: defining and investigating cognitive reserve, brain reserve, and brain maintenance. Alzheimer’s Dement..

[27] Nitrini R (2009). Prevalence of dementia in Latin America: a collaborative study of population-based cohorts. Int. Psychogeriatr..

[28] Quintas JL, Camargos EF, Melo CVS, Nóbrega OT (2017). Influência da escolaridade e da idade em testes cognitivos. Geriatrics, Gerontol. Aging.

[29] Cotrena C, Branco LD, Cardoso CO, Wong CEI, Fonseca RP (2016). The predictive impact of biological and sociocultural factors on executive processing: the role of age, education, and frequency of reading and writing habits. Appl. Neuropsychology: Adult.

[30] Nitrini, R., Barbosa, M. T., Dozzi Brucki, S. M., Yassuda, M. S. & Caramelli, P. Current trends and challenges on dementia management and research in Latin America. *J. Glob. Health* **10**, 010362 (2020). 10.7189/jogh.10.010362 PMC7303806 32566153

[31] Hübner, L. C. et al. Bateria de Avaliação da Linguagem no Envelhecimento – BALE. in *Tarefas de Avaliação Neuropsicológica* (eds Zimmermann, N., Deleaere, F. & Fonseca R. P.) (Memnon, 2019).

[32] Team, R. C. R: a language and environment for statistical computing (2020).

