# Supplementary material for: Reading and writing habits compensate for aging effects in speech connectedness
Source: NPJ Sci Learn. 2022 Jun 8;7:13. doi: 10.1038/s41539-022-00129-8 (PMC9178018; doi:10.1038/s41539-022-00129-8)
Supplement: Supplementary file 1 — Reporting Summary [file 41539_2022_129_MOESM1_ESM.pdf]

nature portfolio

Corresponding author(s): Bárbara Luzia Covatti Malcorra

Last updated by author(s): 2022/05/17

Reporting Summary

Nature Portfolio wishes to improve the reproducibility of the work that we publish. This form provides structure for consistency and transparency in reporting. For further information on Nature Portfolio policies, see our Editorial Policies and the Editorial Policy Checklist.

Statistics

For all statistical analyses, confirm that the following items are present in the figure legend, table legend, main text, or Methods section.

n/a	Confirmed	
☐	☒	The exact sample size (*n*) for each experimental group/condition, given as a discrete number and unit of measurement
☐	☒	A statement on whether measurements were taken from distinct samples or whether the same sample was measured repeatedly
☐	☒	The statistical test(s) used AND whether they are one- or two-sided<br>*Only common tests should be described solely by name; describe more complex techniques in the Methods section.*
☐	☒	A description of all covariates tested
☐	☒	A description of any assumptions or corrections, such as tests of normality and adjustment for multiple comparisons
☐	☒	A full description of the statistical parameters including central tendency (e.g. means) or other basic estimates (e.g. regression coefficient) AND variation (e.g. standard deviation) or associated estimates of uncertainty (e.g. confidence intervals)
☐	☒	For null hypothesis testing, the test statistic (e.g. *F*, *t*, *r*) with confidence intervals, effect sizes, degrees of freedom and *P* value noted<br>*Give P values as exact values whenever suitable.*
☒	☐	For Bayesian analysis, information on the choice of priors and Markov chain Monte Carlo settings
☐	☒	For hierarchical and complex designs, identification of the appropriate level for tests and full reporting of outcomes
☐	☒	Estimates of effect sizes (e.g. Cohen's *d*, Pearson's *r*), indicating how they were calculated

Our web collection on statistics for biologists contains articles on many of the points above.

Software and code

Policy information about availability of computer code

Data collection	Participants performed an oral narrative task based on seven pictures ("The dog story"). The pictures remained available for the participants while telling the story. There was no time limit for the task. Speech samples were audio-recorded and transcribed for analysis. We represented the oral narrative transcriptions as a word-trajectory graph using the Speech Graphs software. To control verbosity, we analyzed the narratives using a moving window of a fixed word length (30 words) with a step of one word. Three connectedness attributes were calculated: (1) repeated edges (RE), defined as the sum of all edges linking the same pair of nodes; (2) the number of nodes in the largest connected component (LCC), defined as the largest set of nodes directly or indirectly linked by some path; and (3) the number of nodes in the largest strongly connected component (LSC), defined as the largest set of nodes directly or indirectly linked by reciprocal paths, so that all the nodes in the component are mutually reachable. We considered RE as short-range and LCC and LSC as long-range speech connectedness.
Data analysis	Since the data were not normally distributed (Shapiro-Wilk test), we used a nonparametric test, Spearman correlations, to assess the association between age, education and frequency of RWH with RE, LCC and LSC. We corrected the significance level by using the Bonferroni test for 3 comparisons (α = .0166). Then, we performed partial correlations and calculated canonical correlations to associate two sets of variables. The first set contained age, education and RWH, while the second set contained the graph attributes: RE, LCC and LSC. Variance inflation factor (VIF) values were within acceptable ranges (<10), suggesting absence of multicollinearity. We used sets of measures with conditioning numbers lower than 30.

For manuscripts utilizing custom algorithms or software that are central to the research but not yet described in published literature, software must be made available to editors and reviewers. We strongly encourage code deposition in a community repository (e.g. GitHub). See the Nature Portfolio guidelines for submitting code & software for further information.

Data

Policy information about availability of data

All manuscripts must include a data availability statement. This statement should provide the following information, where applicable:

- Accession codes, unique identifiers, or web links for publicly available datasets
- A description of any restrictions on data availability
- For clinical datasets or third party data, please ensure that the statement adheres to our policy

All the data are available in Supplementary Table 3.

Field-specific reporting

Please select the one below that is the best fit for your research. If you are not sure, read the appropriate sections before making your selection.

☐ Life sciences ☒ Behavioural & social sciences ☐ Ecological, evolutionary & environmental sciences

For a reference copy of the document with all sections, see nature.com/documents/nr-reporting-summary-flat.pdf

Behavioural & social sciences study design

All studies must disclose on these points even when the disclosure is negative.

Study description	We investigate the association of short and long-range recurrences (speech connectedness) with age, education and reading and writing habits (RWH) in typical aging using an oral narrative production task. Oral narrative transcriptions were represented as word-graphs to measure short and long-range recurrences, using the Speech Graphs sofware (Mota et al., 2012, 2014), a quantitative instrumental.
Research sample	We collected narratives from 118 healthy individuals, predominantly with low educational level and low to middle-low socioeconomic status, in order to mirror low-income countries population, represented by Brazil. Their ages ranged from 51 to 82 (mean = 68.7; standard deviation, SD = 6.44) and education level ranged from 0 to 20 years of formal schooling (mean = 9.99; SD = 5.73). The participants were recruited at community centers, in an urban context in the most southern state in, Brazil.
Sampling strategy	No sample-size calculation was performed.
Data collection	Participants performed an oral narrative task based on seven pictures (“The dog story”). The pictures remained available for the participants while telling the story. There was no time limit for the task. Speech samples were audio-recorded and transcribed for analysis.
Timing	From March 2019 to January 2020.
Data exclusions	No data were excluded.
Non-participation	No participants dropped out.
Randomization	Participants were not allocated into experimental group.

Reporting for specific materials, systems and methods

We require information from authors about some types of materials, experimental systems and methods used in many studies. Here, indicate whether each material, system or method listed is relevant to your study. If you are not sure if a list item applies to your research, read the appropriate section before selecting a response.

Materials & experimental systems

n/a	Involved in the study	
☒	☐	Antibodies
☒	☐	Eukaryotic cell lines
☒	☐	Palaeontology and archaeology
☒	☐	Animals and other organisms
☐	☒	Human research participants
☒	☐	Clinical data
☒	☐	Dual use research of concern

Methods

n/a	Involved in the study	
☒	☐	ChIP-seq
☒	☐	Flow cytometry
☒	☐	MRI-based neuroimaging

Human research participants

Policy information about studies involving human research participants

Population characteristics	We collected narratives from 118 healthy individuals. Their ages ranged from 51 to 82 (mean = 68.7; standard deviation, SD = 6.44) and education level ranged from 0 to 20 years of formal schooling (mean = 9.99; SD = 5.73). All participants had a general cognitive performance within the normal range as measured by the Mini-Mental State Examination (MMSE; mean = 27.25; SD = 2.80).
Recruitment	The participants were recruited at community centers, in an urban context in the most southern state in, Brazil.
Ethics oversight	The study was approved by the Research Ethics Committee at the university where the study was developed under report number 560.073, CAAE registry number 21006913.0.0000.5336. Participation in the study was voluntary, and participants provided their written informed consent before joining the study. Participants were tested individually in a laboratory setting. The participants were recruited at general courses offered for members of the community at the university and at community centers close to it.

Note that full information on the approval of the study protocol must also be provided in the manuscript.
