# Supplementary material for: Reading and writing habits compensate for aging effects in speech connectedness
Source: NPJ Sci Learn. 2022 Jun 8;7:13. doi: 10.1038/s41539-022-00129-8 (PMC9178018; doi:10.1038/s41539-022-00129-8)
Supplement: Supplementary file 2 — Supplementary information [file 41539_2022_129_MOESM2_ESM.docx]

**Supplementary Information for “READING AND WRITING HABITS COMPENSATE FOR AGING EFFECTS IN SPEECH CONNECTEDNESS” by Bárbara L. C. Malcorra, Natalia B. Mota, Janaina Weissheimer, Lucas P. Schilling, Maximiliano A. Wilson, and Lilian C. Hübner**

Supplementary Note 1: Structural speech analysis based on graph theory

The speech graph method was originally proposed to allow the quantification of formal thought disorder associated with schizophrenia disorder^13^. Inspired by the description of symptoms such as “derailment”, “losing of associations”, the idea was to capture the aberrant trajectory of spontaneous narratives by representing each word as a node and the sequence by directed edges. After automatizing the method in 2014^9^, the application for clinical purposes was confirmed in schizophrenia for chronic patients^9^, first-episode patients^15^, and even during prodromal phase^20^, in different cultural contexts and languages^11,20,21^. In general, low connectedness in spontaneous narratives (such as dream reports or picture descriptions) was associated with cognitive decline in schizophrenia, which allowed for an automated diagnosis with high levels of accuracy (around 90%)^9,11,13,15,20,21^.

As these narrative patterns measured by graph attributes are not clearly interpretable, there was an imperative need to understand the typical development of those patterns, and their cognitive basis. For this purpose, children’s spontaneous narratives were collected at school in naturalist experiments. As predicted, connectedness development associated with reduction of short-range recurrences were independently associated with general intelligence, theory of mind abilities, reading performance^10^, and short-term memory performance^12^. The same pattern was also observed during second language acquisition^16^.

To better understand this developmental trajectory, a larger and more diverse population was studied (age range of 2 to 60 years, with educational level of 0 to 20 years)^8^. These data showed the importance of formal education (more than age) for the development of these narrative patterns, with different dynamics for each graph attribute^8^. The further analysis of written texts through 5,000 years of literature revealed a similar dynamics of pattern changes: while a child that starts to read decreases short-range recurrence and increases connectedness in oral narratives, written texts also decreased short-range recurrence while increasing connectedness since Sumerian and ancient Egypt until nowadays literature^18^. Importantly, for the population with psychotic symptoms, none of the sociodemographic variables studied were associated with speech graph attributes^8^, illustrating the need to study those patterns in each context.

Given the association with cognitive decline and development, the method was also applied to other mental disorders, such as ADHD^24^ and dementia^19^, helping to understand the different contribution of semantic and episodic memories in typical and atypical (Alzheimer disease) populations^17^, being recognized by the field as a novel and promising approach for the area of computational psychiatry^22^. Although there is an increasing interest in the application of graph analysis to clinical populations, it is crucial to better understand the typical trajectory of these patterns’ changes, as well as the association with social and environmental variables, enabling a deeper understanding of narrative structures in each different context, considering the most diverse and representative scenario as possible^14,23^.

Supplementary Table 1: Demographic and neuropsychological characteristics of the participants

| **Variable** | **Mean** | **SD** | **SE** |
| --- | --- | --- | --- |
| Age (years) | 68.71 | 6.44 | .59 |
| MMSE | 27.25 | 2.80 | .26 |
| Education (years) | 9.99 | 5.73 | .53 |
| RWH | 12.40 | 6.11 | .56 |
| SES | 24.08 | 6.11 | .57 |
| Sex = Male (%) | 22 (18.6) | | |

Legend: SD = standard deviation; SE = standard errors; MMSE = Mini-Mental State Examination17, with cut-off points established by Laks et al. (2003)18; RWH = reading and writing habits quantified according to the weekly frequency of reading and writing activities with different types of digital and printed texts, with ratings classified as: daily (4 points); a few days a week (3 points); once a week (2 points); rarely (1 point), and never (0 points)19; SES = socioeconomic status (lower SES = 0-16; middle = 17-28; upper-middle = 29-44; upper = 45-100).

Supplementary Table 2: Spearman and partial Spearman correlations for sociodemographic variables and speech graph attributes

|  | RE | LCC | LSC |
| --- | --- | --- | --- |
| ***Age*** | (Rho/ p) | (Rho/ p) | (Rho/ p) |
|  | **.24/ .0087** | **-.23/ .0107** | -.19/ .0445 |
| Adjusted for education | .20/ .0287 | -.18/ .0445 | -.16/ .0802 |
| Adjusted for RWH | .21/ .0226 | -.19/ .0317 | -.15/ .0893 |
|  |  |  |  |
| ***Education*** |  |  |  |
|  | -.22/ .0190 | **.27/ .0034** | .13/ .1682 |
| Adjusted for age | -.17/ .0644 | **.22/ .0138** | .09/ .3320 |
| Adjusted for RWH | -.08/ .3456 | .11/ .2081 | .004/ .9622 |
|  |  |  |  |
| ***RWH*** |  |  |  |
|  | **-.25/ .0061** | **.30/ .0008** | .21/ .0205 |
| Adjusted for age | **-.22/ .0156** | **.27/ .0024** | .18/ .0403 |
| Adjusted for education | -.15/ .0888 | .18/ .0431 | .17/ .0638 |

Legend: Spearman and partial Spearman correlations with significant p-values indicated in bold. Bonferroni correction for 3 comparisons (3 attributes), alpha = .0166.

Supplementary Table 3: Dataset

| Subject | Sex | Age | Education | RWH | SES | MMSE | RE | LCC | LSC |
| --- | --- | --- | --- | --- | --- | --- | --- | --- | --- |
| Sub01 | Female | 63 | 6 | 8 | 14 | 23 | 0.654867 | 23.61947 | 18.9469 |
| Sub02 | Female | 77 | 9 | 8 | 22 | 28 | 1.678082 | 22.32877 | 16.5 |
| Sub03 | Female | 53 | 12 | 13 | 20 | 27 | 0.439024 | 22.7561 | 18.41463 |
| Sub04 | Female | 72 | 3 | 5 | 24 | 28 | 2.826923 | 19.53846 | 17.11538 |
| Sub05 | Female | 59 | 16 | 16 | 21 | 30 | 0.642857 | 24.96429 | 20.42857 |
| Sub06 | Female | 61 | 16 | 22 | 29 | 30 | 0.740426 | 23.42979 | 19.71489 |
| Sub07 | Female | 73 | 16 | 18 | 24 | 25 | 0.61 | 23.58 | 17.62 |
| Sub08 | Female | 56 | 18 | 22 | 29 | 30 | 0.103093 | 25.51546 | 19.54639 |
| Sub09 | Male | 71 | 12 | 16 | 21 | 28 | 1.555556 | 23.97222 | 18.80556 |
| Sub10 | Female | 69 | 16 | 22 | 31 | 30 | 2.458824 | 22.09412 | 19.04706 |
| Sub11 | Male | 65 | 16 | 21 | 38 | 29 | 1.073171 | 23.31707 | 20.17073 |
| Sub12 | Female | 60 | 9 | 9 | 27 | 30 | 0 | 25.48837 | 21.2093 |
| Sub13 | Female | 55 | 0 | 0 |  | 20 | 1.954774 | 21.84422 | 16.44724 |
| Sub14 | Female | 70 | 6 | 21 | 17 | 27 | 0.657895 | 24.5 | 19.81579 |
| Sub15 | Female | 67 | 12 | 15 | 16 | 30 | 1.541667 | 24.04167 | 19.39583 |
| Sub16 | Female | 73 | 8 | 19 | 27 | 30 | 1.238806 | 23.97015 | 20.13433 |
| Sub17 | Female | 61 | 17 | 20 | 34 | 30 | 0.62069 | 24.62069 | 17.37931 |
| Sub18 | Female | 64 | 17 | 18 | 24 | 30 | 1.373333 | 22.62667 | 18.62667 |
| Sub19 | Female | 73 | 18 | 17 | 25 | 30 | 1.085271 | 23.70543 | 18.72868 |
| Sub20 | Female | 71 | 8 | 11 | 22 | 29 | 0.75 | 22.625 | 21.08333 |
| Sub21 | Female | 64 | 18 | 19 | 21 | 30 | 1.121622 | 22.40541 | 19.40541 |
| Sub22 | Female | 79 | 5 | 4 | 24 | 28 | 3.7875 | 20.2 | 16.4125 |
| Sub23 | Female | 73 | 8 | 15 | 15 | 29 | 0.64 | 23.93333 | 17.96 |
| Sub24 | Female | 72 | 4 | 13 | 23 | 30 | 2.025862 | 22.55172 | 18.56034 |
| Sub25 | Female | 62 | 5 | 14 | 27 | 30 | 1.615385 | 22.70769 | 18.66154 |
| Sub26 | Female | 67 | 8 | 13 | 23 | 30 | 1.44186 | 22.11628 | 18.09302 |
| Sub27 | Female | 72 | 4 | 4 | 15 | 25 | 4.797753 | 18.03371 | 16.16854 |
| Sub28 | Male | 78 | 4 | 4 | 27 | 27 | 0.675439 | 23.58772 | 17.77193 |
| Sub29 | Female | 73 | 4 | 11 | 31 | 25 | 1.486726 | 23.48673 | 19.57522 |
| Sub30 | Female | 72 | 9 | 11 | 22 | 23 | 1.053097 | 24.37168 | 17.41593 |
| Sub31 | Female | 66 | 17 | 15 | 25 | 25 | 1.410714 | 23.28571 | 19.89286 |
| Sub32 | Female | 61 | 16 | 22 | 25 | 30 | 1.73913 | 20.97826 | 18.78261 |
| Sub33 | Female | 65 | 20 | 17 | 25 | 27 | 1.789474 | 20.84211 | 17.76316 |
| Sub34 | Female | 63 | 12 | 15 | 23 | 24 | 1.875 | 23.125 | 19.625 |
| Sub35 | Female | 63 | 10 | 13 | 21 | 27 | 0.643836 | 24.09589 | 19.17808 |
| Sub36 | Female | 67 | 16 | 12 | 28 | 27 | 1.362573 | 23.25731 | 18.18129 |
| Sub37 | Female | 65 | 18 | 18 | 27 | 27 | 0.796296 | 24.46296 | 17.33333 |
| Sub38 | Female | 69 | 16 | 17 | 30 | 23 | 1.207547 | 22.84906 | 19.07547 |
| Sub39 | Male | 72 | 14 | 13 | 22 | 28 | 1.666667 | 20.55556 | 18.35714 |
| Sub40 | Male | 75 | 16 | 10 | 35 | 29 | 1.012658 | 25.02532 | 18.12658 |
| Sub41 | Female | 71 | 3 | 9 | 32 | 26 | 1.677419 | 21.93548 | 17.3629 |
| Sub42 | Female | 72 | 19 | 20 | 20 | 28 | 0.795699 | 24.65591 | 18.20968 |
| Sub43 | Female | 72 | 5 | 15 | 21 | 30 | 0.807339 | 23.15596 | 20.11927 |
| Sub44 | Female | 73 | 16 | 18 | 27 | 30 | 2.058333 | 22.38333 | 17.70833 |
| Sub45 | Female | 67 | 13 | 13 | 20 | 28 | 1.977941 | 22.125 | 17.14706 |
| Sub46 | Male | 75 | 10 | 8 |  | 28 | 0.646552 | 24.68103 | 19.01724 |
| Sub47 | Male | 75 | 3 | 2 | 23 | 25 | 1.659574 | 23.82979 | 19.74468 |
| Sub48 | Female | 80 | 1 | 0 | 13 | 16 | 2.518987 | 19.43038 | 16.4557 |
| Sub49 | Female | 60 | 18 | 10 | 28 | 27 | 0.245455 | 25.18182 | 19.19091 |
| Sub50 | Female | 66 | 16 | 2 | 25 | 29 | 1.937888 | 20.86957 | 17.1677 |
| Sub51 | Female | 62 | 16 | 16 | 41 | 29 | 0.958333 | 24.78472 | 19.27778 |
| Sub52 | Male | 78 | 14 | 18 | 25 | 27 | 1.446602 | 23.5534 | 18.1068 |
| Sub53 | Female | 75 | 12 | 14 | 19 | 22 | 3.736111 | 20.63889 | 16.40278 |
| Sub54 | Female | 58 | 3 | 9 | 17 | 22 | 1.538462 | 24.05769 | 21.25 |
| Sub55 | Female | 65 | 6 | 10 | 24 | 27 | 0.876923 | 23.06154 | 19.14615 |
| Sub56 | Female | 64 | 18 | 16 | 38 | 29 | 0.63587 | 25.17935 | 17.38587 |
| Sub57 | Female | 64 | 18 | 19 | 30 | 30 | 1.107595 | 23.32278 | 17.55063 |
| Sub58 | Male | 75 | 8 | 11 | 29 | 28 | 0.393939 | 23.93939 | 20.69697 |
| Sub59 | Female | 71 | 11 | 16 | 29 | 29 | 3.962963 | 20.2037 | 17.75926 |
| Sub60 | Female | 75 | 11 | 13 | 23 | 27 | 1.810345 | 21.93103 | 19.5 |
| Sub61 | Female | 64 | 18 | 16 | 30 | 30 | 2.195652 | 21.54348 | 18.7029 |
| Sub62 | Female | 69 | 16 | 11 | 37 | 28 | 1.603053 | 23.10687 | 14.05344 |
| Sub63 | Female | 76 | 16 | 6 | 18 | 28 | 0.216216 | 25.16216 | 20.09459 |
| Sub64 | Female | 66 | 9 | 19 | 20 | 29 | 1.3125 | 22.9375 | 15.6875 |
| Sub65 | Female | 62 | 15 | 23 | 31 | 30 | 1 | 24.59459 | 20.13514 |
| Sub66 | Female | 51 | 4 | 12 | 17 | 28 | 1.085714 | 23 | 20.65714 |
| Sub67 | Female | 79 | 3 | 15 | 18 | 27 | 1.590361 | 20.16867 | 18.10843 |
| Sub68 | Male | 63 | 0 | 0 |  | 18 | 2.196581 | 20.89744 | 17.41026 |
| Sub69 | Female | 73 | 1 | 20 | 18 | 23 | 1.582822 | 22.33129 | 17.3865 |
| Sub70 | Female | 80 | 3 | 0 | 13 | 26 | 1.474576 | 22.44915 | 18.48305 |
| Sub71 | Female | 67 | 5 | 5 | 18 | 28 | 1.371429 | 23.54286 | 19.11429 |
| Sub72 | Male | 63 | 7 | 10 | 16 | 28 | 1.390977 | 23.53383 | 17.7594 |
| Sub73 | Female | 60 | 7 | 12 | 22 | 27 | 1.019231 | 24.21154 | 18.76923 |
| Sub74 | Male | 75 | 5 | 1 | 24 | 29 | 1.074627 | 22.74627 | 20.9403 |
| Sub75 | Male | 73 | 5 | 5 | 21 | 28 | 3.910714 | 19.30357 | 17.03571 |
| Sub76 | Female | 67 | 4 | 8 | 24 | 29 | 2.967213 | 21.14754 | 16.34426 |
| Sub77 | Male | 62 | 5 | 7 | 19 | 29 | 1.141414 | 22.25253 | 18.90909 |
| Sub78 | Female | 74 | 2 | 1 | 17 | 27 | 1.409836 | 22.19672 | 18.81967 |
| Sub79 | Female | 66 | 3 | 5 | 22 | 22 | 1.376923 | 22.92308 | 17.19231 |
| Sub80 | Male | 72 | 3 | 3 | 22 | 29 | 1.878788 | 22.11111 | 18.41414 |
| Sub81 | Female | 78 | 2 | 5 | 18 | 24 | 3.769231 | 18.07692 | 15.76923 |
| Sub82 | Female | 78 | 14 | 11 | 26 | 29 | 0.90411 | 23.47945 | 20.31507 |
| Sub83 | Female | 67 | 15 | 16 | 29 | 30 | 1.636364 | 23.84091 | 20.52273 |
| Sub84 | Female | 67 | 4 | 15 | 15 | 29 | 1.166667 | 22.5 | 19.7 |
| Sub85 | Female | 68 | 12 | 15 | 20 | 30 | 1 | 23.58824 | 19.91765 |
| Sub86 | Female | 73 | 12 | 9 | 21 | 29 | 1.218391 | 23.09195 | 18.36782 |
| Sub87 | Female | 65 | 13 | 14 | 22 | 28 | 1.864865 | 21.00541 | 16.78919 |
| Sub88 | Female | 68 | 4 | 3 | 25 | 22 | 1.888889 | 22.05556 | 19.38889 |
| Sub89 | Female | 64 | 12 | 22 | 17 | 30 | 0.5 | 26.69792 | 19.08333 |
| Sub90 | Female | 78 | 16 | 26 | 28 | 29 | 1.78 | 21.15 | 17.3 |
| Sub91 | Female | 79 | 11 | 20 | 19 | 27 | 2.424242 | 21.15152 | 18.70707 |
| Sub92 | Female | 73 | 16 | 14 | 26 | 28 | 1.29703 | 22.89109 | 18.82178 |
| Sub93 | Female | 74 | 5 | 12 | 21 | 29 | 3 | 19.9 | 12.5 |
| Sub94 | Male | 64 | 12 | 7 | 32 | 30 | 3.592357 | 19.90446 | 15.87261 |
| Sub95 | Female | 65 | 3 | 11 | 25 | 29 | 2.044643 | 20.20536 | 15.84821 |
| Sub96 | Male | 78 | 4 | 6 | 20 | 27 | 0.962963 | 23.98148 | 17.14815 |
| Sub97 | Male | 67 | 5 | 4 | 23 | 27 | 0.969388 | 21.69388 | 19.07143 |
| Sub98 | Female | 65 | 6 | 10 | 15 | 23 | 1.463415 | 22.12195 | 17.09756 |
| Sub99 | Female | 74 | 5 | 11 | 20 | 23 | 2.041237 | 21.75258 | 17.29897 |
| Sub100 | Male | 72 | 5 | 4 | 24 | 28 | 2.512195 | 21.34146 | 17.31707 |
| Sub101 | Female | 61 | 0 | 11 |  | 22 | 0.875 | 23.98214 | 20.41071 |
| Sub102 | Female | 70 | 2 | 14 | 19 | 25 | 1.77512 | 22.96651 | 16.88517 |
| Sub103 | Female | 65 | 6 | 17 | 18 | 29 | 1.277778 | 23.5463 | 18.0463 |
| Sub104 | Female | 75 | 16 | 18 | 19 | 27 | 1.651515 | 22.55051 | 18.64141 |
| Sub105 | Female | 69 | 14 | 19 | 40 | 28 | 1.206186 | 24.3299 | 19.15464 |
| Sub106 | Female | 63 | 9 | 11 | 28 | 29 | 1.633333 | 22.61111 | 16.5 |
| Sub107 | Female | 71 | 1 | 12 |  | 20 | 2.779412 | 18.99265 | 13.23529 |
| Sub108 | Female | 72 | 16 | 19 | 32 | 28 | 2.215569 | 21.55689 | 17.8503 |
| Sub109 | Male | 63 | 16 | 10 | 32 | 28 | 2.535354 | 20.9798 | 16.93939 |
| Sub110 | Female | 67 | 12 | 24 | 38 | 26 | 0.647619 | 23.88571 | 19.84762 |
| Sub111 | Male | 67 | 5 | 12 | 18 | 25 | 0.269231 | 23.65385 | 19.42308 |
| Sub112 | Female | 62 | 18 | 13 | 35 | 29 | 2.410714 | 21.69643 | 17.17857 |
| Sub113 | Female | 76 | 16 | 16 | 22 | 26 | 3.2 | 20.04 | 17.98667 |
| Sub114 | Female | 60 | 16 | 7 | 32 | 30 | 4.04 | 20.38 | 17.72 |
| Sub115 | Female | 62 | 18 | 15 | 25 | 24 | 1.343949 | 23.8535 | 19.17834 |
| Sub116 | Female | 82 | 8 | 19 | 19 | 29 | 1.141762 | 24.02682 | 18.45977 |
| Sub117 | Female | 82 | 12 | 10 | 30 | 25 | 3.520833 | 18.10417 | 15.94271 |
| Sub118 | Male | 73 | 7 | 3 | 23 | 27 | 1.968085 | 22.18085 | 19.59574 |
